# The Utility of a Brief Web-Based Prevention Intervention as a Universal Approach for Risky Alcohol Use in College Students: Evidence of Moderation by Family History

**DOI:** 10.3389/fpsyg.2018.00747

**Published:** 2018-05-22

**Authors:** Zoe E. Neale, Jessica E. Salvatore, Megan E. Cooke, Jeanne E. Savage, Fazil Aliev, Kristen K. Donovan, Linda C. Hancock, Danielle M. Dick

**Affiliations:** ^1^Department of Psychology, Virginia Commonwealth University, Richmond, VA, United States; ^2^Virginia Institute for Psychiatric and Behavioral Genetics, Virginia Commonwealth University, Richmond, VA, United States; ^3^Laboratory of NeuroGenetics, Department of Psychology and Neuroscience, Duke University, Durham, NC, United States; ^4^Complex Trait Genetics Lab, Center for Neurogenomics and Cognitive Research, Vrije Universiteit Amsterdam, Amsterdam, Netherlands; ^5^Division of Student Affairs, Wellness Resource Center, Virginia Commonwealth University, Richmond, VA, United States; ^6^Department of Human and Molecular Genetics, Virginia Commonwealth University, Richmond, VA, United States; ^7^College Behavioral and Emotional Health Institute, Virginia Commonwealth University, Richmond, VA, United States

**Keywords:** alcohol, college students, family history, BASICS, prevention

## Abstract

**Background:** Alcohol use on college campuses is prevalent and contributes to problems that affect the health, emotional wellbeing, and academic success of college students. Risk factors, such as family history of alcohol problems, predict future alcohol problems, but less is known about their potential impact on intervention effectiveness. The purpose of this study was to examine the effect of an intervention implemented in a non-randomized sample of drinking and non-drinking college freshmen.

**Methods:** Freshmen college students recruited for the intervention study (*n* = 153) completed a web-adaptation of the Brief Alcohol Screening and Intervention for College Students (BASICS) at the start of spring semester. We compared their 30-days post-intervention alcohol initiation, number of drinking days (DAYS), drinks per occasion (DRINKS), maximum drinks in 24 h (MAX24) and alcohol use disorder symptoms (AUDsx) to 151 comparison participants retrospectively matched on demographics and baseline alcohol use behaviors. We also tested baseline DRINKS, DAYS, AUDsx, MAX24, and parental family history (PFH) of alcohol problems as moderators of the effect of the intervention.

**Results:** At follow-up, intervention participants had lower rates of AUDsx than comparison participants, especially among baseline drinkers. Among participants drinking 3+ days/month at baseline, intervention participants showed fewer DAYS at follow-up than the comparison group participants. BASICS was also associated with a decreased likelihood of initiation among baseline non-drinkers. PFH significantly interacted with treatment group, with positive PFH intervention participants reporting significantly fewer AUDsx at follow-up compared to positive PFH comparison participants. We found no evidence for an effect of the intervention on DRINKS or MAX24 in our analyses.

**Conclusions:** Results suggest some indication that novel groups, such as non-drinkers, regular drinkers, and PFH positive students may experience benefits from BASICS. Although conclusions were limited by lack of randomization and short follow-up period, PFH positive and low to moderate drinking groups represent viable targets for future randomized studies.

## Introduction

Heavy alcohol consumption and risky drinking are a prevalent concern on college campuses. Most college students (81%) have tried alcohol in their lifetime, and 35% consumed five or more drinks in one sitting within the prior 2 weeks (Johnston et al., 2016). The probability of experiencing negative outcomes, such as problems with school, emotional health problems, bodily harm or injury, and troubles with the law, is significantly elevated for individuals who engage in risky drinking practices (Hingson et al., 2005). College students are more likely to drink alcohol compared to same-age non-college peers (Johnston et al., 2016), and are more likely to experience clinically significant consequences of their drinking (Slutske et al., 2004; Slutske, 2005). Peer influences, easier access to alcohol, engagement in fraternity/sorority events, and increased independence are some of the factors that can contribute to the pattern of increasing alcohol use evident across the first year of college (Borsari et al., 2007).

In 2002, the National Institute on Alcohol Abuse and Alcoholism issued recommendations for universities to address college student drinking. Yet, 6 years later, Nelson et al. (2010) found that still only 41% of universities required that their students complete alcohol programming. A universal approach to alcohol prevention and intervention allows schools to intervene before violations of university alcohol policies prompt reactionary measures. The need for universal prevention/intervention is supported by the existence of a “prevention paradox” for college student alcohol use, whereby the heaviest drinkers represent a relatively small portion of all alcohol users (Weitzman and Nelson, 2004). Though these individuals are most at risk for alcohol-related harms, they are responsible for only a small portion of the total number of negative consequences that befall college students. Therefore, a focus on delaying the onset of alcohol initiation and preventing the increase of risky drinking behaviors among lower risk students leads to a greater decrease in consequences overall in the college population. Strategies that identify only high-risk individuals for interventions are not sufficient to address the scope of alcohol-related consequences among college students.

Web-based interventions present an opportunity to reach all college students through an efficient and cost-effective avenue. A number of effective, brief motivational interventions (BMIs) have been adapted for use online, such as the Brief Alcohol Screening and Intervention for College Students (BASICS; Dimeff et al., 1999). Web-based adaptations of BASICS consolidate all components of the intervention into a computerized alcohol assessment and electronically delivered Personalized Feedback Profile (PFP). The feedback may include comparisons of one's alcohol use with normative data from their university, as well as additional personalized risk factors, such as family history of alcohol problems, BAC, time it takes for alcohol elimination after a heavy drinking episode (time until sober), caloric intake due to alcohol, and reflection of negative alcohol-related consequences are addressed in the PFP. Each component of the PFP is accompanied by a non-judgmental description of these risk factors as well as strategies for changing behavior when appropriate. Pieces of the feedback profile are adapted to target specific risk factors (e.g., binge drinking, family history of alcohol problems, “pre-gaming”), and the specific language is modified to suit the level of risk expressed by the student. Interventions like BASICS may prove particularly useful for a range of populations due to the customizability of the feedback, relative ease of delivery via the internet, and accessibility compared to other more intensive programs like AlcoholEdu (Cronce and Larimer, 2011; Paschall et al., 2011).

There is ample evidence to support web-based brief interventions like BASICS for at-risk drinkers (Elliott et al., 2008; Carey et al., 2009a), but they have not been as well researched as universal programs. Some studies show support for the use of brief interventions among low-risk or non-drinking college students, while others either show an absence of effect or worse: iatrogenic effects that result in potentially harmful increases in risky drinking behaviors (Werch and Owen, 2002; Bersamin et al., 2007). One study that investigated the effects of a brief, personalized feedback intervention when applied campus-wide as a universal approach to prevention (Palfai et al., 2014) found no differences in alcohol consumption and risky drinking practices between intervention and comparison group participants at follow-up; however, they did find that the intervention was protective against the initiation of alcohol use among baseline non-drinkers. This finding was consistent with results found in two other studies of prevention effects on non-drinking college students (Larimer et al., 2007; Wood et al., 2010). Continued research on the effect of a BASICS program as a universal prevention strategy may provide the evidence needed to replace existing, ineffective strategies for universal alcohol prevention such as education alone (Cronce and Larimer, 2011).

When employing a universal approach to prevention, the drinking characteristics of the sample may vary greatly, ranging from non-drinking to heavy drinking students. Therefore, it is important to examine a range of drinking characteristics to ensure the program is not deemed ineffective due to potential floor effects. In a randomized clinical trial of a universal personalized feedback prevention program, Larimer et al. (2007) found small but significant effects on past month alcohol consumption, but not for alcohol-related negative consequences, possibly due to low endorsement. In this study, the intervention dampened the expected drinking trajectory such that the increases in alcohol consumption observed in the intervention group were significantly smaller than that of the control group. An effective prevention/intervention program with a universal approach might be expected to show maintenance of low levels of use and alcohol-related harms rather than a reduction due to their already low baseline levels. Further, effects of the intervention may depend on the degree to which the desired change or a motivation for change was explicitly addressed in the personalized feedback.

Certain individual risk factors, such as family history (FH) of alcohol problems, may also moderate the effects of web-based interventions. Alcohol use, from initiation to problems, is known to be under genetic influence (Prescott and Kendler, 1999; Kendler et al., 2008; Verhulst et al., 2015). Therefore, college students enter into prevention intervention programs at differing levels of genetic risk. While the identification of the specific genes influencing this predisposition is slowly progressing (Schumann et al., 2016; Clarke et al., 2017), FH remains the strongest predictor that indexes this genetic predisposition (Yan et al., 2014). Furthermore, emerging research of interactions between genotype and intervention suggest that individuals at greatest genetic risk may benefit more from prevention/intervention, making family history (FH) an important potential moderator to be examined (van Ijzendoorn and Bakermans-Kranenburg, 2015). FH captures both genetic and environmental risk for alcohol use disorders, which contributes to the hypothesis that FH may prime an individual for an intervention by virtue of that person having observed firsthand the degree of harm that can result from risky alcohol use (Kendler et al., 2015b). Findings from the few studies that have examined FH as a moderator of intervention effects suggest that college students who self-report positive FH responded better to intervention despite no evidence of baseline differences in substance use or problems (LaBrie et al., 2009; Lee et al., 2010). Exploring moderators of intervention effects, like FH, may help us to uncover otherwise hidden benefits of substance use interventions for specific at-risk groups.

The present study evaluated a web-based alcohol intervention program available for use at a large, public university in U.S. Participants were recruited from the Spit for Science sample, a longitudinal study of genetic and environmental influences on alcohol use, substance use, and emotional health, in which approximately 60% of all freshman students participated within their first semester of college (Dick et al., 2014). In collaboration with university partners in student services, we capitalized an opportunity to examine the existing alcohol prevention program available for students, and study how parental family history, a proxy for genetic risk, might influence outcomes. Freshman college students completed an online version of BASICS at the start of their second semester in college and their post-intervention drinking behaviors (approximately 30 days later) were compared to that of a comparison group that received no intervention. We hypothesized that intervention participants would report significantly fewer drinking days, drinks per occasion, maximum drinks in 24 h, and alcohol use disorder (AUD) symptoms than comparison participants at follow-up. We also hypothesized that the non-drinkers who completed the intervention would be less likely than baseline non-drinkers in the comparison group to report that they had ever consumed alcohol at follow-up. Lastly, we predicted that intervention-associated reductions in drinking would be greater in individuals with baseline higher alcohol use and parental family history of alcohol use problems.

## Materials and methods

### Participants

#### Participant recruitment

Participants were a subsample of the Spit for Science project (Dick et al., 2014), which invited all first-time freshman students age 18 and older to complete an online survey at the start of their first semester. Individuals who completed the survey were invited to provide an optional DNA sample, in order to facilitate the goal of understanding genetic and environmental influences on substance use and emotional health outcomes. We use data from the third cohort of the study, freshmen entering college in the fall of 2013. Of invited participants, 2022 (59%) completed the fall freshman year Spit for Science (S4S) survey. All those who completed the fall survey were invited to participate in a spring follow-up survey administered approximately 6 months after the initial survey. Participants were compensated $10 for completion of the fall survey, and an additional $10 for completion of the spring follow-up survey. Data collection was conducted and maintained using Research Electronic Data Capture (REDCap), a secure, web-based program hosted through the investigators' university (Harris et al., 2009).

At the start of spring semester, a randomly selected subset of students who completed the fall S4S survey (*N* = 797) were invited to participate in a study about the effectiveness of online alcohol educational programming. Potential participants were informed that they would receive $10 in compensation for participating in the study, which required them to complete an online “alcohol education” program. The university where S4S is based has optional, but not mandatory alcohol programming for its students. There were no specific inclusion criteria, which aligned with the goal to evaluate the effectiveness of a brief intervention using a universal (including all students) rather than a targeted approach (limited to heavy drinking or at risk students). However, a group of S4S participants (*N* = 237) that participated in a separate alcohol intervention study was excluded from eligibility (Savage et al., 2015). Following approval from the university's Institutional Review Board and informed consent procedures, a total of 797 randomly selected individuals were invited to participate in order to obtain a target sample of 200 individuals. There were 313 students who expressed interest prior to our cessation of enrollment, and 180 completed the study.

#### Selection of comparison group

**Figure 1:**
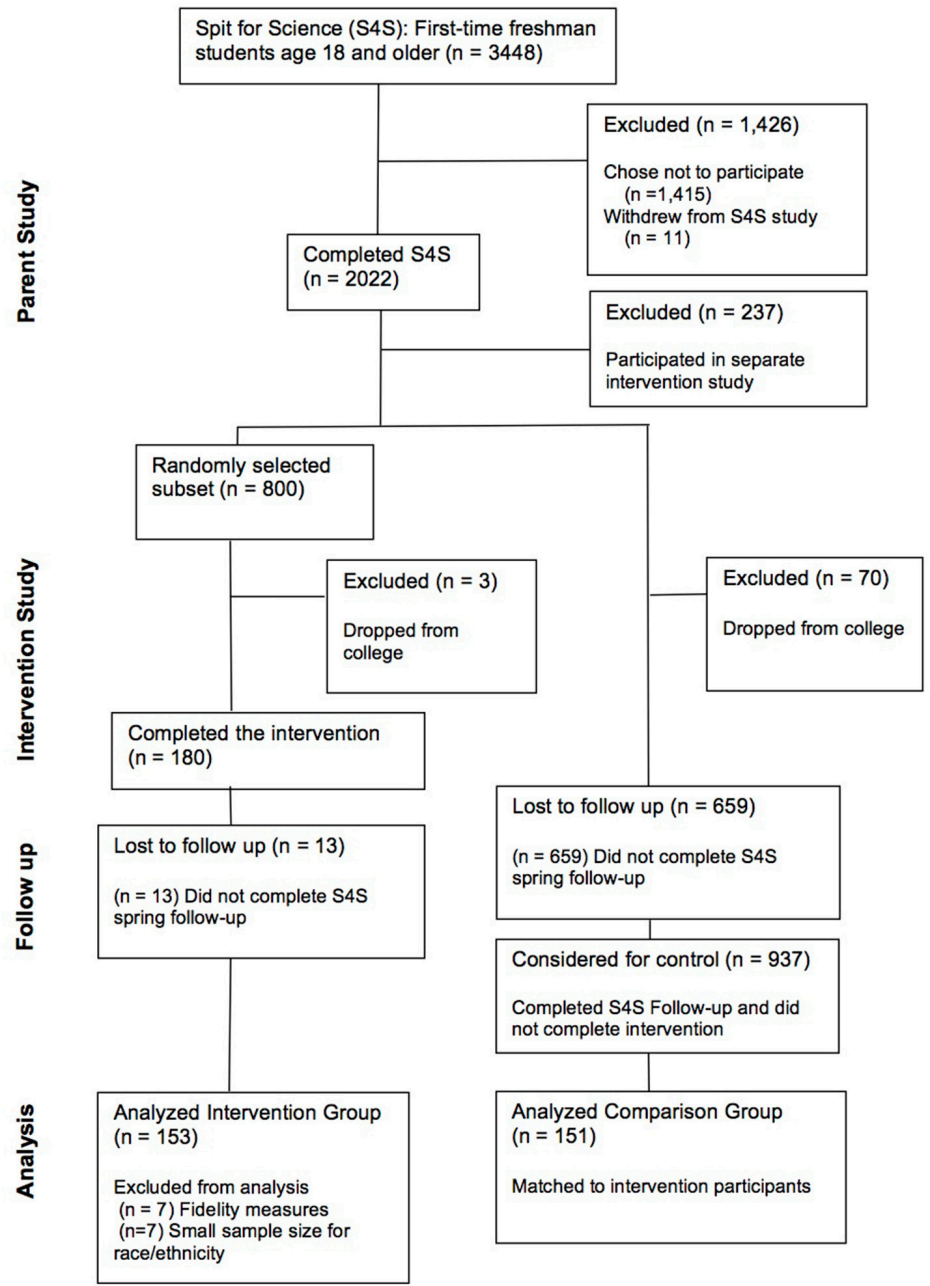
Diagram of participation in parent study, intervention, control, follow up, and inclusion in analyses.

In order to determine if changes in drinking were attributable to the intervention program, we selected a group of comparison participants who completed both the fall and spring S4S surveys, but did not participate in the intervention study. Comparison participants were matched to intervention participants based on ethnicity, sex, and fall alcohol use (number of drinking days per month, typical drinks consumed per occasion, and maximum number of drinks in 24 h). A suitable match was identified for 151 of the 153 intervention participants. There were no significant differences between intervention and comparison participants on the matching variables. Figure 1 provides additional information about recruitment and retention of participants into both the intervention and comparison group.

### Procedure

#### Assessment of alcohol use behaviors

Alcohol use behaviors were assessed for both intervention and comparison participants in the fall (Time 1) and spring (Time 2) semester S4S surveys. We assessed typical number of days drinking per month (DAYS), usual number of drinks per occasion (DRINKS), maximum drinks in 24 h (MAX24), and DSM-5 Alcohol Use Disorder symptoms (AUDsx). DAYS and DRINKS were reported based on past month use at both time points. MAX24 and AUDsx were reported based on lifetime for fall and since beginning college in the spring survey. Ninety-three percent of the intervention study participants completed the S4S alcohol measures at Time 2, which was administered approximately 1 month after the intervention. Comparison participants were only selected if they completed the spring S4S survey.

#### Alcohol prevention intervention program

Upon providing informed consent, intervention participants were presented with a link to complete an online “alcohol education” program (BASICS) (Dimeff et al., 1999; Labrie et al., 2013). In BASICS, participants complete a 15–20 min assessment about their perceptions of and behaviors related to alcohol use and substance use, as well as personal goals for their time in college. This assessment information is then used to create a Personalized Feedback Profile (PFP), which is provided to participants electronically immediately after completing the assessment. Participants received individualized feedback on a number of alcohol risk factors, including the impact of genetic risk for alcohol based on their self-reported family history. The BASICS program data was collected anonymously and utilized only to construct the PFP for the participants' own use.

### Measures

Measures for the key study constructs are detailed below. Participants were allowed to decline to answer any item in the survey by selecting the response “I choose not to answer.”

#### Alcohol initiation

Participants were asked if they had ever consumed a drink of alcohol at least once in their lifetime. Individuals who indicated “no” to the first item were asked to confirm that they had never tried alcohol. Those who confirmed this were identified as baseline non-drinkers, whereas all others were considered baseline drinkers.

#### Alcohol consumption

Two items from the reliable and valid Alcohol Use Disorder Identification Test (AUDIT), a screening measure with established reliability and validity, were used to assess how much (DRINKS) and how often (DAYS) an individual drinks alcohol (Babor and Grant, 1989; Fleming et al., 1991; Demartini and Carey, 2012). Response options for DAYS in the past month were Never (0), Monthly (1), 2–4 times a month (3), 2–3 times a week (10), 4 or more times a week (16). For typical DRINKS in the past month, response options were 1–2 (1.5), 3–4 (3.5), 5–6 (5.5), 7, 8, or 9 (8), and 10 or more (10). Participants also reported the maximum number of alcoholic beverages consumed in a 24-h period (MAX24) in their lifetime at Time 1 and since starting college at Time 2. Non-drinkers were coded as zero for DAYS, DRINKS, and MAX24.

#### Alcohol use disorder symptoms

To measure alcohol problems, 16 items from the Semi-Structured Assessment for the Genetics of Alcoholism (SSAGA) were adapted to measure DSM-5 criteria for AUD (Bucholz et al., 1994). The SSAGA has been established as a reliable measure of problem alcohol use in college students (Slutske et al., 2004). In these items, participants were asked to report how many times they experienced certain alcohol-related problems (e.g., arrested for driving drunk or drunken behavior, problems in love relationship or marriage, drinking interfered with school, work or household duties) and dependence symptoms (e.g., becoming tolerant or experiencing withdrawal symptoms). Response options (“Never,” “1–2 times,” and “3 or more times”) were recoded semi-continuously and summed to create the AUDsx variable. In the fall semester, these items addressed lifetime criteria for AUDs whereas the spring semester addressed the time since initiating college. Non-drinkers were coded as zero for AUDsx.

#### Parental family history of alcohol use problems

Participants indicated in the fall S4S survey whether they believed their mother or father has ever had a problem with alcohol use. Responses were dichotomized to represent the perceived absence (score of 0) or presence (score of 1) of family history of AUDs in either parent. The predictive validity of this measure of family history was established in the same sample by Kendler et al. (2015a).

#### Drinking motives

Four subscales of drinking motives (Social, Coping, Enhancement, Conformation) were measured using the Drinking Motives Questionnaire (Cooper, 1994). Responses were coded such that higher scores conveyed greater motivation to drink for that reason. For more detail on the measurement of drinking motives in the Spit for Science sample, please see Dick et al. (2014).

#### Personality

Participants responded to a subset of items from the Big Five Inventory in the fall S4S survey (John and Srivastava, 1999). Using three items from each of the five subscales, we calculated average scores for Openness, Conscientiousness, Extraversion, Agreeableness, and Neuroticism. Individuals missing greater than 50% of items for each subscale were excluded. For more detail on the measurement of personality in the Spit for Science sample, please see Dick et al. (2014).

#### Impulsivity

Impulsivity was measured at baseline using the UPPS-P Impulsive Behavior Scale (Whiteside and Lynam, 2001; Cyders and Smith, 2007), which has five subscales of impulsivity: Negative Urgency (α = 0.75, Positive Urgency (α = 0.77), Lack of Premeditation (α = 0.75), Lack of Perseverance (α = 0.66), and Sensation Seeking (α = 0.60). Subscales were calculated using three items each, with responses ranging from “Disagree strongly” to “Agree strongly.” Individuals missing greater than 50% of the items for a given subscale were excluded.

#### Fidelity measure

In order to ensure that participants in the intervention group completed the program as instructed, they were asked to answer one fidelity item immediately after completion of the intervention. Participants were asked to enter a code phrase embedded into the last page of their PFP. Individuals who incorrectly entered the correct code phrase were excluded from the analyses due to presumed failure to comply with study procedures (*n* = 7).

#### Demographic covariates

Self-reported race/ethnicity, gender, and age were included as covariates in the analyses. Race/ethnicity was dummy coded using three variables (Asian, Black/African American, Hispanic/Latino), with White serving as the reference group.

### Analytic plan

All analyses were completed using SPSS Statistics, version 23 (SPSS, Chicago, IL). We compared baseline DRINKS, DAYS, AUDsx, and MAX24 between those who did and did not (*n* = 14) complete the Time 2 assessment and observed no significant differences. Therefore, missing data was excluded list-wise. Gender and race/ethnicity were included as covariates in the analyses.

#### Descriptive statistics

We first examined descriptive statistics for all study variables in the intervention and comparison groups, and explored differences between groups using *t*-tests and chi-squared tests. Next, we compared intervention and comparison participants separately according to baseline drinking status (drinkers and non-drinkers). Finally, comparisons between the sample of the present study (intervention and comparison combined), and the overall S4S sample were conducted to evaluate the representativeness of the intervention sample compared to the larger study.

#### Effectiveness of the intervention

Hierarchical multiple regression was used to determine if treatment group (BASICS or comparison) significantly predicted spring semester (Time 2) DRINKS, DAYS, MAX24, and AUDsx after controlling for fall semester (Time 1) drinking, gender, and race/ethnicity. Alcohol variables (DRINKS, DAYS, MAX24, and AUDsx) were transformed using log(alcohol variable + 1) to reduce the effects of non-normality. We also conducted sensitivity analyses by running these hierarchical multiple regression analyses in baseline drinkers and non-drinkers separately.

In non-drinkers, we conducted a logistic regression in order to evaluate the preventive effects of the BASICS Feedback program. An individual's enrollment in either BASICS or the comparison group was used to predict the likelihood that a non-drinker initiated alcohol use in the spring semester.

#### Moderators influencing the effects of the intervention

We hypothesized that individual differences in Time 1 drinking (DRINKS, DAYS, MAX24, and AUDsx) and parental family history of alcohol problems would contribute to the effectiveness of the intervention. To test this hypothesis, Time 1 drinking variables were examined as moderators of the intervention to predict the respective Time 2 drinking variable while accounting for covariates. For example, we tested for interactions between Time 1 DRINKS and treatment group on Time 2 DRINKS, while accounting for age, sex, and race/ethnicity. DRINKS, DAYS, MAX24, and AUDsx were tested in four separate moderator models. Similarly, parental family history was tested as a moderator of the relationship between treatment group and Time 2 alcohol variables. Each alcohol outcome was tested in a separate model, while accounting for the respective Time 1 measure. Regions of significance for significant Time 1 moderators were identified using online resources made freely available by Preacher et al. (2006).

## Result

### Descriptive statistic

Descriptive characteristics for the sample of 304 participants are outlined in Table 1. Matching precluded baseline differences on all alcohol variables. Of note, there were no significant differences between the intervention and comparison group on the proportion reporting a parental history of problems with alcohol, even though participants were not matched on this factor. We also examined drinking motives, personality, and impulsivity to determine if there were any differences between the two groups on factors that might motivate a student to participate in an intervention. We observed no significant differences between intervention and comparison participants on any of the subscales for drinking motives, personality, or impulsivity.

**Table 1:** Descriptive characteristics for intervention and comparison group participants.

**Variable**	**Intervention Group *n* = 153**	**Comparison Group *n* = 151**	***t*/*z*/χ^2^ (*p*)**
Age [mean (SD)]	18.40 (0.35)	18.45 (0.32)	−1.24 (0.22)
**SEX [*n* (%)]**			
Female	105 (69)	104 (69)	0.05 (0.96)
**RACE/ETHNICITY (*n* [%])**			
Asian	34 (22)	34 (23)	−0.06 (0.95)
Black/African American	32 (21)	32 (21)	−0.06 (0.95)
Hispanic/Latino	10 (7)	8 (5)	0.46 (0.65)
White	77 (50)	77 (51)	−0.12 (0.91)
**INITIATED ALCOHOL USE (*n* [%])**			
Fall drinker	86 (56.2)	85 (56.3)	<0.01 (0.99)
Spring drinker	110 (71.9)	118 (78.1)	1.74 (0.19)
**Alcohol use (mean [SD])**			
Time 1 DAYS	1.63 (3.40)	1.37 (2.80)	0.75 (0.46)
Time 2 DAYS	1.91 (2.91)	1.98 (3.00)	−0.21 (0.83)
Time 1 DRINKS	1.66 (2.40)	1.71 (2.44)	−1.90 (0.85)
Time 2 DRINKS	2.19 (2.40)	2.47 (2.45)	−1.02 (0.31)
Time 1 MAX24	4.34 (5.71)	3.80 (5.05)	−0.84 (0.40)
Time 2 MAX24	5.47 (5.97)	6.10 (5.68)	0.89 (0.37)
Time 1 AUDsx	1.71 (2.70)	1.90 (3.10)	−0.58 (0.56)
Time 2 AUDsx	2.23 (2.73)	2.60 (3.05)	−1.00 (0.32)
**PARENTAL FAMILY HISTORY OF ALCOHOL PROBLEMS (*n* [%])**			
Mother, Yes	10 (6.5)	6 (4.0)	1.00 (0.32)
Father, Yes	27 (17.6)	28 (18.5)	−0.20 (0.84)
Either parent, Yes	29 (19.0)	30 (19.9)	−0.20 (0.84)
**DRINKING MOTIVES (MEAN [SD])**			
Social motives	2.81 (0.89)	2.96 (0.69)	1.17 (0.24)
Coping motives	1.56 (0.80)	1.73 (0.92)	1.26 (0.21)
Enhancement motives	2.73 (0.90)	2.83 (0.90)	0.69 (0.49)
Social conformity motives	1.29 (0.57)	1.36 (0.60)	0.79 (0.43)
**PERSONALITY (MEAN [SD])**			
Extraversion	10.10 (2.98)	10.51 (2.75)	1.22 (0.23)
Conscientiousness	13.40 (1.95)	13.42 (1.86)	0.10 (0.92)
Agreeableness	12.31 (2.11)	12.36 (1.97)	0.24 (0.81)
Neuroticism	8.33 (2.76)	8.18 (2.70)	−0.49 (0.63)
Openness	12.56 (1.94)	12.47 (2.10)	−0.37 (0.71)
**IMPULSIVITY (MEAN [SD])**			
Negative urgency	2.12 (0.80)	2.16 (0.71)	0.46 (0.65)
Lack of premeditation	1.77 (0.60)	1.73 (0.51)	−0.72 (0.48)
Lack of perseverance	1.65 (0.55)	1.63 (0.51)	−0.37 (0.71)
Sensation seeking	2.83 (0.69)	2.95 (0.68)	1.61 (0.12)
Positive urgency	1.94 (0.71)	2.01 (0.73)	0.81 (0.42)

*Total N = 304. Age is calculated in years for Time 1 using date of birth. DAYS, days drinking in the past 30 days; DRINKS, past 30 days drinks per occasion; AUDsx, Alcohol Use Disorder symptoms; MAX24, maximum drinks in 24 h. DAYS and DRINKS per occasion were reported on past 30 days use. AUDsx and MAX24 were reported on lifetime at Time 1 and since starting college at Time 2*.

Across time, both the comparison and intervention groups showed significant increases in all alcohol variables from Time 1 to Time 2; however, there were no significant differences between groups at either time point. Table 2 presents *t*-test results for comparisons of Time 1 and Time 2 DRINKS, DAYS, MAX24, and AUDsx between intervention and comparison participants, separated by baseline drinking status (drinker or non-drinker). The mean number of Time 2 AUDsx was significantly lower among baseline drinkers in the intervention group compared to the comparison group.

**Table 2:** Comparison of alcohol variables and parental family history between intervention and comparison participants separated by baseline drinking status.

	**Baseline Drinkers** ***N** = * **171 (56.3%)**			**Baseline Non-drinkers** ***N** = * **128 (42.1%)**		
**Variable**	**Intervention *n* = 86**	**Comparison *n* = 85**	***t* (*p*)**	**Intervention *n* = 64**	**Comparison *n* = 64**	***t* (*p*)**
Time 1 DAYS	2.87 (4.10)	2.42 (3.37)	0.78 (0.44)	–	–	–
Time 2 DAYS	2.91 (3.33)	3.19 (3.51)	−0.54 (0.59)	0.62 (1.44)	0.43 (0.80)	0.92 (0.36)
Time 1 DRINKS	2.94 (2.54)	3.15 (2.53)	−0.54 (0.59)	–	–	–
Time 2 DRINKS	3.32 (2.48)	3.70 (2.29)	−1.02 (0.31)	0.75 (1.22)	0.90 (1.63)	0.59 (0.56)
Time 1 MAX24	7.55 (5.64)	6.86 (4.90)	−0.89 (0.38)	–	–	–
Time 2 MAX24	8.00 (5.98)	8.86 (5.49)	0.99 (0.32)	1.8 (2.93)	2.36 (3.67)	0.88 (0.38)
Time 1 AUDsx	3.56 (2.84)	3.97 (3.35)	−0.79 (0.43)	–	–	–
Time 2 AUDsx	3.29 (2.85)	4.41 (3.23)	−2.39 (0.02)^*^	1.11 (1.71)	1.02 (1.50)	0.29 (0.78)

Time 1 variables were measured in the fall semester of freshman year, and Time 2 were measured in the spring semester of freshman year. DAYS, days drinking in the past 30 days; DRINKS, past 30 days drinks per occasion; MAX24, maximum drinks in 24 h; AUDsx, Alcohol Use Disorder symptoms.

* signifies p-value below 0.05.

** *signifies p-value below 0.01*.

In comparison to the overall S4S sample from which the present sample was recruited, the S4S sample was significantly higher on Time 1 and Time 2 DRINKS, DAYS, MAX24, and AUDsx (*t* ranging from 2.21 to 8.25, *p*-values 0.003 to >0.001). The overall S4S sample and subset for this study did not differ on parental family history of alcohol problems.

### Effectiveness of the intervention

#### Full sample

There was no evidence that treatment group was significantly associated with Time 2 DAYS, DRINKS, or MAX24 after accounting for the demographic covariates and Time 1 drinking variables (Table 3). However, there was a negative association between treatment group and Time 2 AUDsx, β = −0.10, *t*_(294)_ = −2.13, *p* = 0.03, indicating that completing BASICS was associated with lower levels of Time 2 AUDsx compared to comparison participants.

**Table 3:** Results of hierarchical multiple regression: testing the effect of treatment group on Time 2 DAYS, DRINKS, MAX24, and AUDsx in the full sample, baseline drinkers, and baseline non-drinkers.

	**Full Sample** ***N*** = **291**		**Baseline Drinkers** ***N*** = **165**		**Baseline Non-Drinkers** ***N*** = **126**	
	**β**	***t* (*p*)**	**β**	***t* (*p*)**	**β**	***t* (*p*)**
**TIME 2 DAYS**						
Asian	−0.10	−2.04 (0.04^*^)	−0.16	−2.08 (0.04^*^)	−0.04	−0.37 (0.71)
Black/African American	0.02	0.44 (0.66)	−0.01	−0.17 (0.87)	0.02	0.20 (0.84)
Hispanic/Latino	−0.05	−1.07 (0.29)	−0.07	−0.90 (0.37)	−0.05	−0.51 (0.61)
Gender (male = 1)	−0.02	−0.40 (0.69)	0.07	0.94 (0.35)	−0.14	−1.45 (0.15)
Time 1 DAYS	0.58	11.9 (<0.001^**^)	0.38	5.0 (<0.001^**^)	–	–
Treatment group (BASICS = 1)	−0.01	−0.26 (0.80)	−0.05	−0.77 (0.45)	0.07	0.76 (0.45)
**TIME 2 DRINKS**						
Asian	−0.04	−0.96 (0.34)	−0.04	−0.51 (0.61)	−0.05	−0.50 (0.615)
Black/African American	0.01	0.28 (0.78)	−0.01	−0.08 (0.94)	0.03	0.28 (0.78)
Hispanic/Latino	−0.02	−0.40 (0.69)	−0.02	−0.26 (0.80)	−0.05	−0.48 (0.63)
Gender (male = 1)	−0.01	−0.17 (0.86)	0.08	1.13 (0.26)	−0.08	−0.79 (0.43)
Time 1 DRINKS	0.69	15.45 (<0.001^**^)	0.57	8.21 (<.001^**^)	–	–
Treatment group (BASICS = 1)	−0.03	−0.64 (0.52)	−0.07	−1.05 (0.30)	−0.01	−0.10 (0.92)
**TIME 2 MAX24**						
Asian	−0.05	−0.68 (0.50)	−0.13	−1.83 (0.07)	−0.09	−0.89 (0.38)
Black/African American	0.03	0.43 (0.67)	0.03	0.44 (0.66)	−0.01	−0.09 (0.93)
Hispanic/Latino	0.06	0.72 (0.47)	0.05	0.68 (0.50)	−0.01	−0.12 (0.92)
Gender (male = 1)	0.18	2.25 (0.03^*^)	−0.13	−1.79 (0.08)	−0.01	−0.06 (0.95)
Time 1 MAX24	0.44	5.44 (<0.001^**^)	0.39	5.30 (<0.001^**^)	–	–
Treatment group (BASICS = 1)	−0.06	−0.80 (0.43)	−1.3	−1.84 (0.07)	−0.08	−0.80 (0.43)
**TIME 2 AUDSX**						
Asian	−0.08	−1.53 (0.13)	−0.07	−0.89 (0.38)	−0.13	−1.18 (0.24)
Black/African American	−0.03	−0.58 (0.56)	−0.10	−1.14 (0.26)	−0.02	−0.15 (0.88)
Hispanic/Latino	−0.04	−0.73 (0.47)	−0.07	−0.81 (0.42)	−0.04	−0.43 (0.67)
Gender (male = 1)	0.01	0.14 (0.89)	0.03	0.33 (0.74)	−0.04	−0.40 (0.69)
Time 1 AUDsx	0.68	12.15 (<0.001^**^)	0.28	3.55 (0.001^**^)	–	–
Treatment group (BASICS = 1)	−0.10	−2.13 (0.03^*^)	−0.27	−3.42 (0.001^**^)	0.01	0.11 (0.91)

Time 1 variables were measured in the fall semester of freshman year, and Time 2 were measured in the spring semester of freshman year. DAYS, days drinking in the past 30 days; DRINKS, past 30 days drinks per occasion; MAX24, maximum drinks in 24 h; AUDsx, Alcohol Use Disorder symptoms. For race, White was used as the reference group.

* signifies p-value below 0.05.

** *signifies p-value below 0.01*.

#### Baseline drinkers

Sensitivity analyses among those who endorsed a history of alcohol consumption at baseline showed similar results. Completing BASICS predicted fewer Time 2 AUDsx [β = −0.27, *t*_(135)_ = −3.42, *p* = 0.001], but there was no evidence for an association with Time 2 DRINKS, DAYS or MAX24. Treatment group explained an additional 4.3% of the variance in Time 2 AUDsx over and above the effect of Time 1 AUDsx, race/ethnicity, and gender, Δ*F*_(1, 135)_ = 11.70, *p* = 0.001.

#### Baseline non-drinkers

We used logistic regression to determine if non-drinkers who completed BASICS (*n* = 64) were less likely to report ever consuming alcohol in the spring semester than baseline non-drinkers in the comparison group (*n* = 62). Results indicate that individuals who completed BASICS were 91% less likely to report ever consuming alcohol at Time 2, $\chi_{\left(5\right)}^{2}$ = 18.86, *p* = 0.002, while controlling for gender and race/ethnicity. Beyond the effect on trying alcohol, there was no evidence that, among baseline non-drinkers, treatment group predicted Time 2 DAYS, DRINKS, MAX24, or AUDsx.

### Moderators influencing the effects of the intervention

#### Baseline alcohol use characteristics

The effect of treatment group on Time 2 DAYS was moderated by Time 1 DAYS [Δ*R*^2^ = 0.011, β = −0.16, *t*_(283)_ = −2.24, *p* = 0.03], such that those with higher levels of Time 1 DAYS who completed BASICS showed lower rates of Time 2 DAYS compared to comparison participants. Simple slopes analyses used to identify the region of significance for this moderation effect indicated that it falls between 2.65 and 16.98 Time 1 DAYS per month. This suggests that BASICS participants who reported drinking about three or more days per month at Time 1 demonstrated significantly fewer Time 2 drinking days than comparison group participants with comparable drinking frequency at Time 1.

For DRINKS, there was no evidence that Time 1 DRINKS moderated the association between BASICS and Time 2 DRINKS. We also found no interaction between Time 1 AUDsx and BASICS on Time 2 AUDsx, or Time 1 MAX24 and BASICS on Time 2 MAX24. This suggests that the effect of BASICS on Time 2 DRINKS, MAX24, and AUDsx does not vary at different levels of baseline drinking in our sample.

#### Parental family history

**Figure 2:**
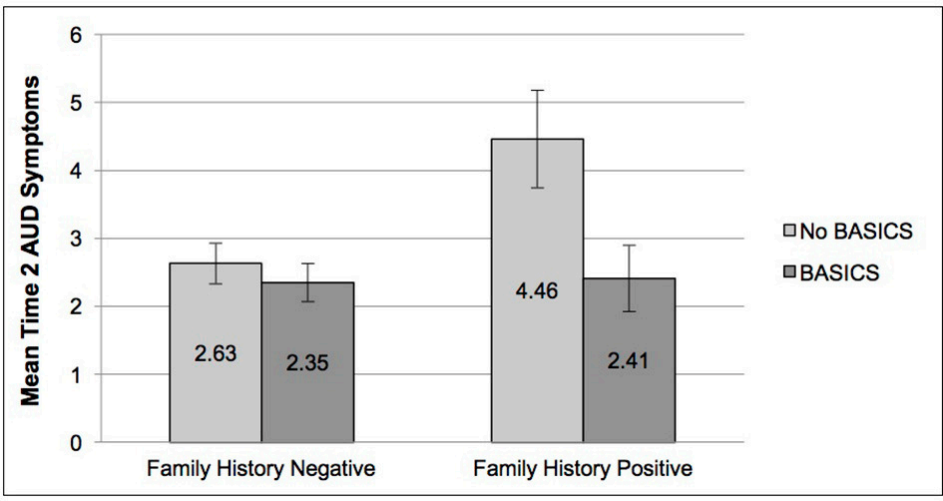
Time 2 alcohol use disorder symptoms as a function of parental family history and intervention status.

As shown in Table 4, we found that PFH significantly moderated the relationship between BASICS and Time 2 AUDsx, β = −0.15, *t*_(211)_ = −1.95, *p* = 0.05. Figure 2 shows the direction of this effect was such that PFH+ individuals who completed BASICS reported significantly fewer Time 2 AUDsx than those who did not complete BASICS. There was no evidence of an interaction between BASICS and PFH on Time 2 DAYS, DRINKS, or MAX24.

**Table 4:** Results of moderation analyses: interaction between treatment group and parental family history on Time 2 AUDsx.

	**β**	***t* (*p*)**	***R^2^***	***ΔR^2^***
**TIME 2 AUDsx**				
*Step 1*			0.40	–
Asian	−0.08	−1.30 (0.20)		
Black/African American	−0.004	−0.07 (0.95)		
Hispanic/Latino	−0.06	−1.13 (0.26)		
Gender (male = 1)	0.002	0.04 (0.97)		
Time 1 AUDsx	0.61	11.12 (>0.001^**^)		
*Step 2*			0.41	0.012
Parental Family History (PFH)	−0.01	−0.10 (0.92)		
Treatment group	−0.12	−2.05 (0.04^*^)		
*Step 3*			0.42	0.011
Treatment group^*^PFH	−0.15	−1.95 (0.05^*^)		

Time 1 AUDsx was measured in the fall semester of freshman year, and Time 2 was measured in the spring semester of freshman year. AUDsx, Alcohol Use Disorder symptoms. For race, White was used as the reference group. For Parental Family History, 0 = no parental family history of alcohol problems, 1 = positive parental family history of alcohol problems.

* signifies p-value below 0.05.

** *signifies p-value below 0.01*.

## Discussion

The present study examined the effect of a brief, web-based preventive intervention on alcohol consumption and alcohol use disorder symptoms in a non-randomized sample of diverse college freshmen. A wide range of drinking patterns were represented in the sample, from non-drinkers to heavy drinkers, so as to better understand the effectiveness of BASICS employed as a universal program for all college students.

### Summary of findings

Our primary question, whether BASICS was effective in the short-term as a universal prevention intervention, was addressed by testing the effect of BASICS on alcohol consumption and alcohol use disorder (AUD) symptoms across the whole sample, and then separately among baseline drinkers and nondrinkers. Both intervention and comparison group participants significantly increased alcohol consumption and AUD symptoms from fall to spring semester, which is consistent with other research that has shown alcohol use increases across the first year of college (Baer et al., 2001). We found no support for the hypothesis that completion of BASICS was associated with fewer drinking days, drinks per occasion, or maximum drinks in 24 h in the spring semester (Time 2). However, for AUD symptoms, we found that BASICS was associated with fewer symptoms in the spring semester in the whole sample, an effect that was driven by baseline drinkers. This finding was not significant for the baseline non-drinking group. There were some benefits to completing the intervention for non-drinkers, though. Non-drinkers who completed BASICS were less likely to initiate alcohol consumption in the spring semester.

Variations in baseline characteristics (drinking days, drinks per occasion, maximum drinks in 24 h, AUD symptoms, and parental family history) were examined as moderators of intervention effectiveness. Higher rates of Time 1 days drinking days were associated with fewer Time 2 days drinking among those who participated in BASICS compared to comparison participants. We observed no evidence for baseline drinks per occasion, maximum drinks in 24 h, or AUD symptoms as moderators of the association between treatment group and Time 2 outcomes. Parental family history significantly moderated the association between treatment group and spring AUD symptoms, such that PFH+ individuals who completed BASICS had fewer AUD symptoms compared to PFH+ comparison participants.

### Effectiveness of the intervention

As hypothesized, we observed significantly lower AUD symptoms among those who completed the BASICS intervention. We also expected to see significantly fewer drinking days, drinks per occasion, and maximum drinks in 24 h in the spring semester among those who completed BASICS, but this was not supported in our findings. Our results on drinking days and drinks per occasion are consistent with Palfai et al.'s (2014) study of a universal alcohol intervention with Personalized Normative Feedback, in which the authors also found no significant differences in alcohol consumption between their intervention and comparison participants at 5 months post-intervention. However, our findings for AUD symptoms contrast with findings from Palfai et al., who found no significant differences on their measure of negative consequences. It may be that the measure of AUD symptoms assessed more common experiences than the measure of negative consequences, which Palfai et al. reported were relatively low on average in their sample.

Sensitivity analyses among baseline non-drinkers, showed that the intervention was associated with lower rates of starting to drink alcohol at follow-up. This provides further support for BASICS as a tool to prevent initiation of alcohol use among non-drinking students, a finding that was also observed in Palfai et al.'s study. Though this shows promise for BASICS as a universal prevention intervention, it is also important to recall that the messaging for BASICS is designed to resonate with heavier drinking students. Therefore, brief interventions with content tailored to better reflect the perspectives of non-drinking students might provide additional benefits beyond the preventive effects found in our study.

Among baseline drinkers, we observed an effect of the intervention on AUD symptoms but not alcohol consumption. Other research has found reductions in alcohol frequency (Labrie et al., 2013) and quantity (Saitz et al., 2007) among students who complete web-based BASICS. However, these studies typically focus on high-risk and heavy drinking samples, whereas our analyses included all students who had initiated any alcohol use. Studies of college students with more heterogeneous drinking behaviors, such as ours, have produced mixed results. Bersamin et al. (2007) observed beneficial effects on consumption and negative consequences among regular drinkers, but not those who had not consumed alcohol in the month prior to attending college. Several other studies produced no effects or only improved knowledge, beliefs and intention (Meier, 1988; Reis et al., 2000; Sharmer, 2001; Kypri and Mcanally, 2005; Moore et al., 2005). The presence of an effect on AUD symptoms in the absence of differences in consumption in our sample may be due in part to the harm-reduction approach of BASICS. Students may have practiced protective strategies learned through BASICS that reduced the likelihood of negative consequences without reducing their level of consumption (e.g., alternating with alcoholic beverages with water, eating before drinking, and planning safe transport home).

### Moderators of intervention effectiveness

Our finding that the relationship between BASICS and post-intervention drinking days differed as a function of baseline rates of alcohol use provides some support for the hypothesis that baseline drinking characteristics play a role in the effectiveness of the intervention. These findings fit with existing literature that has shown alcohol interventions in college students can interrupt the expected drinking trajectory such that the degree of increase in alcohol use is much less than would be expected (Larimer et al., 2007). We also found support for parental family history as a moderator of the intervention's effect on spring AUD symptoms among baseline drinkers and the whole sample. Although there has been little research on the role of family history of AUDs in college student interventions, our results are consistent with one study that found reductions in alcohol consumption were amplified among family history positive individuals who completed a female-specific motivational enhancement program (LaBrie et al., 2009). Results from a randomized clinical trial of a brief, web-based intervention for marijuana use among college students also support greater reductions in marijuana use for individuals with a family history of a drug problem (Lee et al., 2010). Although we did not observe any effect of parental family history on alcohol consumption, this pattern of results is consistent with meta-analytic findings that family history has a greater impact on alcohol consequences and AUD symptoms than consumption in college students (Elliott et al., 2012). Our findings, in conjunction with this existing literature, suggest that more research on the association between family history and intervention response is warranted.

The overall pattern of our moderation analyses suggests that BASICS was more effective for those with an elevated risk profile: higher baseline alcohol frequency and a parental family history of alcohol problems. This pattern indicates that BASICS operated as designed, as a selective program for individuals who are at increased risk for problems or have already experienced problems. However, the interaction effect for drinking days included individuals who endorsed approximately 3 or more drinking days per month, which indicates that BASICS also had a positive effect on post-intervention drinking days for individuals who were drinking with regularity at baseline in the fall semester, not just heavy drinking students. In addition, BASICS was protective against initiation of alcohol use for individuals who were non-drinkers when they completed the intervention. Considered together, these findings suggest that BASICS may be beneficial for more heterogeneous student populations in the short-term. Future research should explore the longevity of these findings, particularly as effects from computerized BASICS programs have been shown to diminish over time (Carey et al., 2009b, 2012).

### Study strengths, limitations, and future directions

#### Strengths

The sample was relatively large and diverse in terms of both demographics and alcohol characteristics. The inclusion criteria did not limit participation to heavy drinkers, which allowed for a more normative range of drinking experiences to be represented. The study took advantage of existing resources at the university, in that the web-based BASICS program was already available for use by the students. Further, the ongoing longitudinal nature of the Spit for Science project will provide this study with annual follow-up of alcohol use outcomes while minimizing participant burden.

#### Limitations

In addition to the strengths described, the results of our study are best understood in the context of several limitations. First, each alcohol use outcome was examined separately in independent models, but existing research has shown that these factors are correlated. However, each outcome (drinks per occasion, drinking days, maximum drinks, and AUD symptoms) is targeted through the content in web-based BASICS, which warrants testing these dimensions separately. Second, moderation analyses require large samples, and our study may have been underpowered to detect some of the hypothesized interaction effects. By traditional guidelines (Cohen, 1988), all tests were adequately powered to discern a small effect. However, authors of a meta-analytic review of moderation found that the average effect size observed in moderation analyses is very small, and suggested that researchers reduce the anticipated interaction effect size in power analyses to reflect these findings (Aguinis et al., 2005).

Third, the study employed a non-randomized design, and although we observed no significant differences at baseline between intervention and comparison participants on matched and non-matched variables, there is a possibility that volunteering to complete the intervention or assessment reactivity may have influenced the results. Fourth, the intervention sample was significantly lower on all alcohol variables than the larger, Spit for Science from which they were recruited. It is possible that heavier drinking students may be less inclined to participate in universal alcohol prevention. Non-completion of universal prevention has been shown to predict alcohol-related harms, thus additional effort may be required to intervene upon risky alcohol behaviors in such individuals (Abrams et al., 2011). Lastly, the study relied on self-report data, and although every effort was made to ensure to that participants understood the data they provided was confidential, there may have been concerns about reporting underage drinking. Self-report data is also vulnerable to measurement error, as it relies on the participant's ability to accurately recall the information in question.

#### Future directions

This study contributes to our understanding of the utility of a brief, web-based universal approach to alcohol prevention and intervention for college students. Our results encourage further research on the effectiveness of web-based BASICS program with non-drinkers, low-risk drinkers, and individuals with a parental family history of alcohol problems. In addition, continued measurement of the intervention effects on alcohol use using Spit for Science longitudinal follow-up surveys may clarify whether the short-term effects observed in our study persist across the college years. Prior meta-analytic research on BASICS has indicated that effects benefits dissipate in long-term follow-up (Carey et al., 2009b). Lastly, our research indicates that in addition to being a risk factor for future alcohol problems, parental family history may operate as a moderator of the effect of a brief motivational intervention. Future studies should explore this and other potential moderators of interventions, particularly those for which there is support for their association with the development of alcohol alcohol problems (e.g., impulsivity).

## Conclusion

The findings from this study suggest that our universal application of a web-based BASICS intervention produced differential effects across our sample. Completing the intervention was associated with significantly fewer spring semester AUD symptoms, an effect found largely among baseline drinkers. We also found that among those drinking regularly at baseline (approximately three or more days per month), intervention participants reported significantly fewer spring drinking days than comparison participants. Among non-drinkers, completing BASICS greatly reduced the likelihood of initiating alcohol use in the spring semester. Intervention participants with a positive parental family history of alcohol problems showed greater reductions in AUD symptoms than comparison participants with a parental family history. However, across our analyses we observed no intervention effects on typical drinks consumed or maximum drinks consumed in 24 h. Additional tailoring of intervention content for lower-risk college students and individuals with a parental family history of alcohol problems may further improve outcomes for these groups. More research on specific components of interventions that facilitate change in behavior may be helpful for untangling the question of what works best for each individual risk profile.

## Ethics statement

This study was carried out in accordance with the recommendations of the Virginia Commonwealth University (VCU) Institutional Review Board (IRB) with informed consent from all subjects. The protocol was approved by the IRB at VCU.

## Author contributions

Under the mentorship of DD, ZN managed data collection, conducted the literature review for the introduction and discussion sections, conducted the statistical analyses, and wrote the manuscript. DD, LH, and KD collaborated to determine the research design and obtain funding. JesS, JeaS, and MC provided support on analyses and interpretation of results. FA created the matched comparison group. DD, JesS, JeaS, and MC reviewed and contributed written feedback to all parts of the manuscript. All authors have approved the final version for publication.

### Conflict of interest statement

The authors declare that the research was conducted in the absence of any commercial or financial relationships that could be construed as a potential conflict of interest. The reviewer, DP, and handling Editor declared their shared affiliation.
